# Stereotactic body radiotherapy: No longer a special procedure?

**DOI:** 10.1002/acm2.12853

**Published:** 2020-03-16

**Authors:** Justin D. Gagneur, Andrew Godley, Yi Rong

**Affiliations:** ^1^ Department of Radiation Oncology Mayo Clinic Arizona Phoenix AZ USA; ^2^ Department of Radiation Oncology University of Texas Southwestern Dallas TX USA; ^3^ Department of Radiation Oncology University of California‐Davis Cancer Center Sacramento CA USA

## 1 INTRODUCTION

Centers for Medicare & Medicaid Services (CMS) proposed a reform from fee‐for‐service payment to a prospective, episode‐based payment model for radiation oncology.^1^ Consensus between industry and radiation oncology providers is that the radiation oncology alternative payment model (RO‐APM) would result in a decline in Medicare payments, and thus require participants to adopt more cost‐effective treatment regimens. A previous Parallel‐Opposed editorial debated the benefits and detriments of RO‐APM to the medical physics profession, and it also revealed a new issue for discussion: is there a need to redefine physicists’ roles regarding increased utilization of hypofractionation and/or stereotactic body radiotherapy (SBRT).^2^ SBRT has been considered a special procedure that requires physicists’ supervision for individual treatment planning and delivery. A decade ago, Benedict et al. published the Task Group (TG) 101 report addressing the specific definition of SBRT along with the requirements and resources in establishing a SBRT program.^3^ In the 10‐yr mark since TG101 was published, clinics are generally familiar with the SBRT planning process, the technology is more reliable, and delivery workflow is well established, which begs the question: do we still consider SBRT a special procedure and need to apply the same level of physicist oversight and attention for every case? Herein, we invited two experts debating the proposition that “SBRT is no longer a special procedure”, with Mr. Justin Gagneur arguing for it, and Dr. Andrew Godley arguing against it.

Mr. Justin Gagneur is a Medical Physicist and Program Director of the Medical Physics Residency in the Department of Radiation Oncology at Mayo Clinic in Arizona, Phoenix. He has been focused on applying statistical process control in the clinic and bringing leadership techniques and ideas from corporate America to clinical Medical Physics. His current AAPM participation includes serving as a member of the Working Group on External Beam Quality Assurance (WGEBQA).

Dr. Andrew Godley received his Ph.D. in High Energy Physics from the University of Sydney in 2001 and contributed to experiments at CERN and Fermilab until 2007 when he trained in medical physics at the Medical College of Wisconsin. Dr. Godley spent 7 yrs at the Cleveland Clinic and a year at the Miami Cancer Institute before becoming the Director of Clinical Physics at University of Texas (UT) Southwestern in 2019. Dr Godley has been deeply involved SBRT planning and delivery throughout his medical physics career. He has been the physics representative on the Cleveland and UT safety and process improvement committees.

## 2 OPENING STATEMENTS

### 2.1 Justin D. Gagneur

Stereotactic body radiotherapy was once confined to academic centers and their affiliates. It was the purview of clinical trials that regulated its planning and delivery. Body frames and complex secondary coordinate systems demanded the undivided attention of the physicist covering the case. It was difficult, time consuming, and novel uncharted territory. At this time Physicists played a vital role in ensuring plan quality and delivery accuracy of SBRT treatments. Now SBRT has become an unremarkable part of most if not all radiation oncology departments. Its planning and delivery, aside from the dose per fraction, is often no different than standard fractionation cases. In the initial stage of introducing and implementing a special procedure to a clinic, physicists still supervise dosimetrists and therapists in terms of treatment plan creation and dose delivery. In addition, physicists design system checks, safety precautions, and standards that allow the special case to become routine. Given these realities, when can a special treatment type be considered routine and no longer demand physics’ personal supervision?

AAPM Medical Physics Practice Guideline (MPPG) 9.a^4^ lays out when and how a Physicist should supervise the treatments. These guidelines are incorporated verbatim into ACR technical standards and ASTRO’s Safety is No Accident.^5^ Specifically, personal supervision is required for the first fraction and then direct supervision (i.e., immediately available) suffices for subsequent fractions for any type of SBRT treatment. These specific requirements are from AAPM TG‐101 which was first published in 2010.^3^

At that time high‐precision IGRT systems and patient safety systems, such as LIDAR arrays, camera angles designed for patient monitoring, and three‐dimensional machine models for collision prevention were not commonplace in the clinic. The evolution of our treatment delivery machines and the evolution of the physicist’s role within the clinic have largely addressed the concerns stated in TG‐101. In particular, I would argue that our therapist colleagues, not us, are best qualified to align the patient via IGRT and to monitor the patient for movement and potential collisions.

A close read of MPPG 9.a gives the physicist an important charge that has not been incorporated by the ACR and ASTRO. Section 4.B.2 “Process commissioning and clinical implementation” subsection 5 states that “The SOP (standard operating procedure) should clearly describe the professional supervision requirements for each SRS‐SBRT treatment type”. Therein lies the sticky wicket. Not all SBRT treatments are created equal. For instance, a clinic may make the determination that an SBRT treatment involving phase gating is considered special and therefore requires personal supervision for every treatment. Likewise, an upper lobe lung lesion involving minimal respiratory motion may not require personal supervision at all, even for the first treatment. Site‐specific resources, expertise, and procedures should inform any decision made about physics supervision.

AAPM has given us the tools to aid us in making these decisions. TG‐100^6^ and the practice guidelines provide a framework that allows the qualified medical physicist (QMP) along with departmental leadership, dosimetrists, therapists, and administrators to make safe decisions about physics supervision. Developing standard operating procedures must be a group interdisciplinary effort. No one knows better than an experienced dosimetrist how to make the best plan or a therapist how to safely treat a patient, just like only an administrator can effectively speak to the risk that an institution is willing to take on.

All of this segues well into how our field is changing. The Radiation Oncology Alternative Payment Model (RO‐APM) is likely to increase the amount of SBRT treatments that a clinic performs to maximize payments. I ask what increases a physicist’s value more; standing at a LINAC personally supervising a case or using the time to partner with administrators and leaders within the department to redefine the role that physicists play in increasing patient safety and overall satisfaction? As Dr. Pawlicki advocated for in Decembers’ parallel opposed editorial,^2^ our value should not be defined by what billing codes are associated with our professional presence but by what we as a profession do best: innovating, educating, and increasing patient safety.

Therefore, SBRT is no longer a special procedure when the representatives from all parts of the department come together with the QMP to develop the standards of practices that allow for a safe SBRT treatment to occur without the personnel supervision of the QMP. How this manifests will be different for each clinic, but I think a hard look should be taken at SBRT supervision and how best to maximize both physicists’ value and patient safety.

### 2.2 Andrew Godley

It is incongruous to think that SBRT is no longer a special procedure, when in truth SBRT is a more technical procedure than ever before. SBRT provides an ablative dose, which is very effective but at risk of causing great harm.^7^ Technology, applied by the physicist, maximizes the effectiveness of SBRT, while minimizing the risks. There is considerable new technology combined in SBRT treatments: surface guidance, marker tracking, breath control, and real‐time imaging. Couple this with efforts to utilize less immobilization and decreased margins to limit toxicity and the opportunities for mistreatment have clearly escalated. RO‐APM^1^ favors SBRT, but not by treating SBRT as a standard delivery. This would impede the true goal of RO‐APM: cost effectiveness. With expanding indications and evolving technology, SBRT will remain a special procedure *and* require physics oversight.

There is a constant flow of new technology into our field, and by the time one technology has been fully integrated and transferred to the therapists (e.g., CBCT), another has entered the field (MR‐guided therapy). It is always this latest technology that we wish to apply to SBRT. These technologies, however, provide numerous challenges, including interoperability,^8^ retraining, and added complexity. If SBRT stayed with frames, x‐ray imaging, and 3D conformal techniques, it could fall off our radar and become a standard therapy. To expand to more patients and disease sites though, SBRT has continued to evolve and remains at the forefront of technology. Thus, the physicist will always be integral to every aspect of SBRT.

The physicist has a holistic view of radiation treatment. We understand the simulation, immobilization, planning, imaging, delivery, and summation of delivered dose. As a special procedure, SBRT is an opportunity for physicists to apply the principles of MedPhys 3.0^9^ and demonstrate the direct benefits we provide to patients. By treating SBRT as a special procedure with the physicist available, delays and errors are avoided. Having an involved and readily available physicist alleviates the need to page and wait when difficulty with the equipment, a mistake in set up or planning, or difference in anatomy since the plan was created arises. Upon eventual arrival, an uninvolved physicist would then have to understand the problem, what occurred, and what has already been tried. These delays may cause the patient to move, requiring re‐imaging or restarting the whole procedure. Conversely, an involved physicist would be present at the simulation and know the patient set up, either developed or checked the plan, and immediately be able to aid the therapists and physician to safely treat the patient. Physicist presences also prevent the therapists from being overwhelmed by the number of screens they need to monitor due to the additional imaging modalities used.

Advanced imaging is used in SBRT to monitor internal motion, reducing treatment margins. Whereas before the emphasis of SBRT was on patient setup, strongly in the realm of the therapist, now we also rely on signals from potentially multiple imaging systems that may not directly interface with the radiation delivery system. Sources could be magnetic resonance,^10^ optical,^11^ ultrasound,^12^ thermal, or breathing traces,^13^ beyond the x‐ray–based imaging that therapists are well versed in. Interpretation of these systems, the appearance of images and warnings that they give, requires a physicist. Confirmation that the correct patient, plan, and isocenter are loaded on auxiliary systems requires a physicist. Assurance that the imaging has provided and applied the correct shifts, and that the delivery is being tracked or gated correctly requires a physicist. Technology evolves so quickly that by the time the therapists are comfortable, an update has arrived. Updates introduce new workflows or even just a change in appearance that again require a physicist. The physicist involvement however does not reduce the cost effectiveness of SBRT.

The emphasis of the RO‐APM is to reduce costs. SBRT does this by reducing the number of treatments a patient receives, not by reducing the quality of those treatments. SBRT is the exact solution the RO‐APM needs. The fact that more treatments will be SBRT does not reduce the special nature of each individual SBRT treatment. Switching to an RO‐APM should not strip SBRT of special procedure status. Motion management, immobilization, and imaging have allowed us to reduce planning margins and reduce toxicity to nearby organs.^14^ The effect of this is that SBRT cannot tolerate any uncertainty. Unsupervised SBRT would instead increase the overall cost of care due to toxicity management and recurrences, and detract from the cost‐effectiveness goal of the RO‐APM.

Stereotactic body radiotherapy is always at the leading edge of technology and innovation. This keeps SBRT as a special procedure, requiring physicist involvement. SBRT is a cost‐effective special procedure. The next step in SBRT will not be reducing oversight, but rather reducing the number of fractions, permanently cementing SBRT as a special procedure.

## 3 REBUTTAL

### 3.1 Justin D. Gagneur

Andrew makes a compelling argument for physicist involvement in all aspects of SBRT treatments. In essence we are arguing what level of supervision is appropriate for SBRT. As he elegantly states, how treatment technology is integrated into the clinic by physicists and then fully transferred to the therapists is of the upmost importance. Any new technology implementation should be carefully and personally supervised by the physicist. However, when a technology has been fully transferred to the therapist does it continue to demand personal supervision? I would argue that at that point a threshold has been crossed into the realm of direct supervision (i.e., immediately available).

As capital budget cycles bring in new technologies every 2–10 yrs, the QMP along with physician leadership, dosimetrists, therapists, and administrators should evaluate the workflows and develop a plan to efficiently and safely transfer that technology to the therapist. The supervision of SBRT can then be granular and ever evolving. As Andrew rightly states, our field is in a constant state of technological, medical, and procedural evolution. Our supervision of SBRT should be constantly assessed and interrogated to make sure we are maximizing both physicists’ value and patient safety.

Our field has a tendency to refer to SBRT as a single monolithic procedure; much like the general public tends to refer to cancer as a singular disease. But we are physicists and we know better. The demands of a particular SBRT procedure can be as different as a poorly differentiated head and neck cancer versus a stage 1 DCIS of the breast. As MPPG 9.a states each SBRT treatment type should have its own standard operating procedure and a clearly described level of supervision. Some types of SBRT treatment will continue to demand personal supervision and heavy physics involvement at every step of the planning and delivery process. However, as technology is transferred to the treating therapists and standard operating procedures are developed and strictly adhered to; the procedure may no longer demand personal supervision from physics only direct or general supervision.

In this paradigm, Andrew and I are no longer of opposed viewpoints. Rather we are arguing that SBRT has varied supervision needs depending on many complex and interwoven factors. Before advocating for a type of SBRT treatment to no longer be personally supervised, standard operating procedures must be implemented and all technology being used must be fully transferred to the therapist. In this way we will do what we as physicist do best: innovate, educate, and increase patient safety.

### 3.2 Andrew Godley

First I would like to start with the points from Mr Gagneur I agree with. SBRT is no longer in the realm of academic centers, and should be used with care by all centers. It is correct to consider each SBRT treatment individually and determine what amount of oversight is required, that sounds like a special procedure though. Lastly, physicists should put in place system checks for SBRT delivery, this is stronger than relying on humans, be they physicists or therapists. The more hard stops and interlocks the safer the treatment will be. It is unfortunate, however, that the whole SBRT workflow has too many steps to be designed completely error proof.

We should not, however, conflate SBRT becoming more popular with becoming safer. This is where the danger lies, when procedures become routine. A clinic’s original SBRT procedure that was once strictly followed deteriorates, therapists switch machines, therapists train other therapists, and steps get left out as their importance is lost. Once the supervision has been removed from one “safe” site (upper lobe lung), it quickly erodes from the others. The therapists will question why page the physicist for one SBRT site, and not for the other, or why page for gating but not breath hold. Safety relies on every patient, every time mentality.

The concerns of TG 101^3^ have not disappeared since 2010. Moreover, additional concerns have been added due to the new technology I described. A new Task Group is not required to know a physicist is needed to aid with these, or to let administrators and insurance know SBRT is still a special procedure.

Stereotactic body radiotherapy remaining a special procedure is not related to billing codes or keeping physicists active. SBRT is cost effective, for the patient and the clinic, in any model. Physicists are not there to fill time or sign a document. They are there to oversee the procedure they implemented and help when problems inevitably arise with the complex steps of SBRT. While some cases may have fewer steps, they all deliver a high dose per fraction, after which no correction can be made. To leave to administrators to balance the risk and cost of mistreatment is terrifying. The risk we should be accepting is zero, and we should do everything we can to achieve this.

To misquote Mr Gagneur “SBRT is unremarkable, aside from its dose per fraction.” And that is exactly the point, because where are we putting this dose per fraction? Next to the esophagus, bronchus, rib, duodenum, spinal cord, bladder, and rectal walls. If SBRT becomes a conventional treatment with an ablative dose our patients will bear the burden.

## CONFLICTS OF INTEREST

The authors declare no conflicts of interest.

## References

[1] Proposed Radiation Oncology (RO) Model . 2019 https://www.cms.gov/newsroom/fact-sheets/proposed-radiation-oncology-ro-model, accessed November 2019.

[2] Pawlicki T , Ford E , Rong Y . Radiation oncology alternative payment model to medical physics profession: more benefits than detriments. J Appl Clin Med Phys. 2019;20:6–9. 10.1002/acm2.12782 PMC6909120 31833638

[3] Benedict SH , Yenice KM , Followill D , et al. Stereotactic body radiation therapy: the report of AAPM Task Group 101. Med Phys. 2010;37:4078–4101. 20879569 10.1118/1.3438081

[4] Halvorsen PH , Cirino E , Das IJ , et al. AAPM‐RSS medical physics practice guideline 9.a. for SRS‐SBRT. J Appl Clin Med Phys. 2017;18:10–21. 10.1002/acm2.12146 PMC5874865 28786239

[5] Safety is no accident: A framework for quality radiation oncology and care. http://www.astro.org/safetyisnoaccident.

[6] Huq MS , Fraass BA , Dunscombe PB , et al. The report of Task Group 100 of the AAPM: application of risk analysis methods to radiation therapy quality management. Med Phys. 2016;43:4209–4262. 27370140 10.1118/1.4947547 PMC4985013

[7] Timmerman R . Stereotactic body radiation therapy for inoperable early stage lung cancer. JAMA. 2010;303:1070–1076. 20233825 10.1001/jama.2010.261 PMC2907644

[8] Rengan R , Curran B , Able C , et al. Addressing connectivity issues: the integrating the healthcare enterprise‐radiation oncology (IHE‐RO) initiative. Pract Radiat Oncol. 2011;1:226–231. 24674000 10.1016/j.prro.2011.06.016

[9] What is Medical Physics 3.0? https://w3.aapm.org/medphys30/index.php.

[10] Rosenberg SA , Henke LE , Shaverdian N , et al. A multi‐Institutional experience of MR‐guided liver stereotactic body radiation therapy. Adv Radiat Oncol. 2019;4:142–149. 30706022 10.1016/j.adro.2018.08.005 PMC6349638

[11] Hoisak JDP , Pawlicki T . The role of optical surface imaging systems in radiation therapy. Semin Radiat Oncol. 2018;28:185–193. 29933878 10.1016/j.semradonc.2018.02.003

[12] Su L , Iordachita I , Zhang Y , et al. Feasibility study of ultrasound imaging for stereotactic body radiation therapy with active breathing coordinator in pancreatic cancer. J Appl Clin Med Phys. 2017;18:84–96. 28574192 10.1002/acm2.12100 PMC5529166

[13] Lu L , Diaconu C , Djemil T , et al. Intra‐ and inter‐fractional liver and lung tumor motions treated with SBRT under active breathing control. J Appl Clin Med Phys. 2017;19:39–45. 29152835 10.1002/acm2.12220 PMC5768033

[14] Molitoris JK , Diwanji T , Snider JW 3rd , et al. Optimizing immobilization, margins, and imaging for lung stereotactic body radiation therapy. Lung Transl Cancer Res. 2019;8:24–31. 10.21037/tlcr.2018.09.25 PMC6351403 30788232

